# Law and the radiologist, socio-legal perspectives in radiology practice, lex radiologica: Fundamental commandments of radiology practice in the times that be

**Published:** 2008-08

**Authors:** BB Thukral

**Affiliations:** Department of Radiodiagnosis and Imaging, Vardhman Mahavir Medical College and Safdarjung Hospital, New Delhi - 110 029, India

*No civilization…would ever have been possible without a framework of stability, to provide the wherein for the flux of change. Foremost among the stabilizing factors, more enduring than customs, manners, and traditions, are the legal systems that regulate our life in the world and our daily affairs with each other*.

– Hannah Arendt (political philosopher) in *Crises of the Republic*.

Man is a social animal. Ever since the Old Stone Age, humans have formed communes to live in groups so that they could be safe from the elements inimical to their safety. As man evolved, so did the rules that governed society. The great Indian sage Manu wrote a treatise titled *Manu Smriti*(1500 BC), which was a social code for those times. The guiding principle of the *Manu Smriti* was that people in a civilized society must follow a set of rules and regulations that serve the best interests of its members. This basic doctrine was also the spirit behind the ancient pious thought of *bahujana hitaya, bahujana sukhaya*. The objective of such essays was simply to guard against anarchy and lawlessness in human society and to make life more organized and orderly.

In modern times, with the progress made on various fronts, it has become necessary to formulate laws, rules, and regulations so that all sections of society can live in peace and harmony and take full advantage of the developments in the scientific field. Over the eons, small groups of people have evolved into larger clusters, which have further organized themselves into nation states with well-defined boundaries. Each nation has formulated its own set of rules and regulations in the form of a ‘constitution,’ which is the supreme governing directive that helps those in power run a country and provide a safe and orderly life to its citizens. The constitution defines the rights and duties of the citizens and the executive. Every citizen of the nation state is expected to express and owe allegiance to the constitution and to follow the rules set out therein.

The radiologist is also a citizen and therefore bound by the constitution of the country. To safeguard the interests of the people at large, the government has framed rules and regulations for doctors of all disciplines. These include mandatory registration of medical professionals with the Medical Council of India (MCI) or with the medical councils of their respective states before practicing in any discipline of medicine. Doctors registered with the MCI have to follow the rules, regulations, and ethical guidelines set out by the council.

The doctor - patient relationship requires confidentiality and trust. The doctor has to maintain records of medicolegal cases and assist the judiciary and police in carrying out mandatory legal procedures and provisions. In recent times, the onus on the radiologist to maintain records has increased tremendously, particularly after the Pre-Conception and Pre-Natal Sex Determination (PC-PNDT) Act as well as the Consumer Protection Act (CPA). Under the CPA, the patient has been presumed to be a customer/consumer and the doctor has been equated with a service provider. This exposes medical doctors to the risk of litigation and compensation claims. Sometimes cases are filed for frivolous reasons: serving as outlets for the frustration felt after the loss of a loved one or simply with the intent of extracting money from the doctor, which may be at the behest of unscrupulous lawyers, often termed ambulance chasers. At other times, within the medical profession, professional rivalry may cause one doctor to instigate litigation against another. This violates the basic tenets of medical practice and no doubt makes Hippocrates turn in his grave in the island of Cos.

In order to save/protect oneself from being on the wrong side of the law, the practicing doctor must be prudent; patients' records must be maintained with the utmost sincerity and the doctor must be careful to give well-considered and guarded opinions. Further, it is suggested:

1. Records should be very clear and must be regularly updated. Along with the history of the current illness, details of past illnesses, addictions, treatment received, and occupational history should be recorded even if the answers are in the negative. Any fact not taken on record cannot be proved in the court of law unless it is in the written format. When the final impression is recorded, it would be prudent to mention that the opinion given is subject to review in the light of any new clinical information.
2. Written and informed consent is mandatory. The doctor should explain in detail all the procedures and the risks associated with any investigation/treatment and a written consent/acknowledgement should be obtained. The patient should always be offered a choice of all available, better and safer alternatives, howsoever expensive they may be.
3. The radiologist should avoid jumping the gun. For example, a radiologist conducting an ultrasound examination on a patient should not be in a rush to comment on every finding seen on the monitor and immediately pronounce a diagnosis, nor should he make any remark against another radiologist or clinician. If there are any differences of opinion on the findings or diagnosis, they should be communicated in the proper way and at the appropriate time.
4. Male radiologists examining a female patient should ensure that there is a chaperone or a female attendant or a close relative present throughout the procedure as a precaution against any subsequent charges of misbehavior or even rape.
5. One must always remember the Hippocratic Oath while interacting with patients. The ideals enshrined in the oath should be the guiding principle for all the doctors. At no time should these be forgotten or disregarded.
6. In the unfortunate event of being dragged to a court of law, one should not panic or lose confidence. The facts and findings of the case should be studied in detail and the defense should be prepared accordingly. Since the judges are not trained in medicine it is often difficult for them to understand the intricacies in the field, especially so in the specialized field of radiological diagnostic imaging. Under such circumstances, one must request referral of the case to the MCI/respective state medical council, which is the right body to decide if there has been any negligence on the part of the doctor. Following such a referral, the medical council is expected to appoint an investigating committee of renowned and senior doctors who are experts in the particular discipline to which the case pertains. The opinion of the committee is sought and disciplinary action is then taken by the medical council based on the merits of the case. The opinion of the medical council is also respected by the courts. However, if the action of the medical council is contested in the court by the complainant, the defendant should always demand the opinion of experts from higher institutes of learning. The court has to appoint senior experts from the medical colleges/higher institutions of learning who have more experience than the defendant. The defendant can demand this, as the opinion of a junior person with less experience than the defendant, is not tenable in the eyes of the law.
7. There should be interdepartmental meetings or interactions in the best interests of the patients, and professional egos should be kept in check.

To avoid the inconvenience of facing the law, one must take care to always be on its right side. Radiologists must establish rapport with the patients and their attendants and strive to always maintain good relations with them as well as with fellow radiologists and clinicians. All those concerned with the care of the patient must be kept informed about the developments that occur in the condition of the patient and the likely outcomes of any investigation/treatment. A doctor should also update his/her knowledge on a regular basis and keep abreast of the advances in his/her field. There is an ever-increasing reliance on radiological investigations by the clinicians. Hence, the role of the radiologist is assuming greater importance with each passing day.

All the necessary infrastructure and supportive equipment should be kept in readiness in the radiography/CT/MRI/interventional suite. The personnel working in these areas should be well versed with preventive precautions and know how to institute resuscitative measures in the eventuality of any mishap or allergic reaction. It is very well said that ‘forewarned is forearmed’; it is also said that ‘if it can happen, it will happen’. There is no room for any laxity and no substitute for sincerity in any field, and this is especially true in the field of medicine, as we are dealing with precious human life. These suggestions must not only be put into practice but should also be allowed to percolate down to the next generation of budding radiologists.

